# Elevated Level of Wnt5a Protein in Localized Prostate Cancer Tissue Is Associated with Better Outcome

**DOI:** 10.1371/journal.pone.0026539

**Published:** 2011-10-24

**Authors:** Azharuddin Sajid Syed Khaja, Leszek Helczynski, Anders Edsjö, Roy Ehrnström, Anna Lindgren, David Ulmert, Tommy Andersson, Anders Bjartell

**Affiliations:** 1 Division of Urological Cancers, Department of Clinical Sciences, Lund University, Skåne University Hospital, Malmö, Sweden; 2 Center for Molecular Pathology, Lund University, Skåne University Hospital, Malmö, Sweden; 3 Division of Cell and Experimental Pathology, Department of Laboratory Medicine, Lund University, Skåne University Hospital, Malmö, Sweden; 4 University and Regional Laboratories Region Skåne, Clinical Pathology, Malmö, Sweden; 5 Department of Mathematical Statistics, Center for Mathematical Sciences, Lund University, Lund, Sweden; Huntsman Cancer Institute, University of Utah, United States of America

## Abstract

**Background:**

Wnt5a is a non-canonical secreted glycoprotein of the Wnt family that plays an important role in cancer development and progression. Previous studies report that Wnt5a is upregulated in prostate cancer and suggested that Wnt5a affects migration and invasion of prostate tumor cell. This study aimed to evaluate the prognostic value of Wnt5a protein expression in prostate cancer tissue and its potential to predict outcome after radical prostatectomy in patients with localized prostate cancer.

**Methodology and Results:**

Immunohistochemical analysis of a tissue microarray containing prostate specimens of 503 patients with localized prostate cancer showed significantly higher Wnt5a protein expression in cancer compared to benign cores from the same patients (p<0.0001). Patients with high expression of Wnt5a protein had significantly better outcome in terms of time to biochemical recurrence compared to patients with low expression levels (p = 0.001, 95%CI 1.361–3.570, Hazard's ratio 2.204). A combination of high Wnt5a expression with low levels of Ki-67 or androgen receptor expression had even better outcome compared to all other groups. Furthermore, we found that Wnt5a expression significantly correlated with VEGF and with Ki-67 and androgen receptor expression, although not highly significant. In vitro, we demonstrated that recombinant Wnt5a decreased invasion of 22Rv1 and DU145 cells and that siRNA knockdown of endogenous Wnt5a protein led to increased invasion of 22Rv1 and LNCaP cells.

**Conclusion:**

We demonstrate that preserved overexpression of Wnt5a protein in patients with localized prostate cancer predicts a favorable outcome after surgery. This finding together with our in vitro data demonstrating the ability of Wnt5a to impair the invasive properties of prostate cancer cells, suggests a tumor suppressing effect of Wnt5a in localized prostate cancer. These results indicate that Wnt5a can be used as a predictive marker and that it also is a plausible therapeutic target for treatment of localized prostate cancer.

## Introduction

Prostate cancer (PCa) is the leading cancer affecting men of all races and the second most leading cause of death in developed countries [1]. Androgens and the androgen receptor (AR) play critical roles not only in normal development, growth and function of the prostate gland but also in carcinogenesis and progression of PCa [2]. Initially, PCa cells are commonly AR dependent for their growth and survival, and hence respond to androgen deprivation therapy (ADT), but in later stages PCa cells become androgen-insensitive, and fatal castration-resistant prostate cancer (CRPC) develops [3]. The molecular mechanisms responsible for transition into CRPC are poorly understood, however, the most consistent change associated with castration-resistant growth in global gene expression profiles of PCa xenografts was an increase in the AR mRNA levels [4]. Increased expression of AR is considered to be a key feature of CRPC and it has been demonstrated as a consequence of either mutation or amplification of AR or by increased expression caused by deregulated growth factors or various co-regulators [5]. Although we have access to prognostic factors in PCa, including Gleason grade, TNM stage, surgical margin status and serum PSA levels, there is an urgent need to identify novel biomarkers, which can significantly improve, either alone or in combination of other biomarkers, our ability to predict outcome in PCa patients. Previous studies have suggested a possible relationship between AR and Wnt-β-catenin signaling pathways during the development and progression of PCa [6], [7].

Recently, attention has been drawn to the role of Wnt proteins and Wnt signaling in PCa. The name Wnt comes from “wingless-related MMTV integration site” and was originally suggested by Nusse and co-workers in 1991 [8]. Wnt proteins constitute a family of nineteen secreted glycoproteins that play important roles during development and in cell fate specification, cell migration and cell polarity [9], [10]. Wnt proteins can be classified into at least two subfamilies; canonical Wnts that promote β-catenin-mediated transcription and non-canonical Wnts. Wnt signaling occur in an auto- or paracrine fashion through binding of secreted Wnt molecules to seven transmembrane Frizzled receptor proteins (Fz) in the absence or presence of co-receptors such as LRP 5/6 and ROR [10]. Several Wnt signaling components have also been implicated in genesis of human cancers; overexpression of Wnt-1 was observed in mammary epithelial adenocarcinoma [11] and in several PCa cell lines and PCa tissues. Wnt-1 expression positively correlated with Gleason score, β-catenin and with serum PSA levels [12]. In addition, based on the determination of Wnt5a mRNA levels in prostate tumors it has been suggested that abnormal expression of the non-canonical Wnt5a is involved in PCa [13].

Wnt5a, one of the most studied non-canonical Wnts, is an essential Wnt protein in inducing and controlling the Wnt/planar cell polarity (PCP) and the Wnt/Ca^2+^ pathways [14], [15]. In addition, Wnt5a has not only been demonstrated to counteract the Wnt/β-catenin pathway but also, in specific contexts, to activate this pathway [16]. The possibility of Wnt5a to induce different downstream signaling events can at least in part explain the presence of reports suggesting an ambiguous nature of Wnt5a; having either a tumor suppressor or tumor promoting function depending on context and tumor type [16]. Previous studies have shown that Wnt5a is downregulated in certain malignancies including colorectal cancer (protein expression) [17], neuroblastoma (mRNA levels) [18], invasive ductal breast carcinomas (protein expression) [19], [20] and leukemias (mRNA levels) [21], indicating a tumor suppressing effect of Wnt5a. Interestingly, other reports have instead suggested an oncogenic effect of Wnt5a primarily based on an upregulation in breast cancer cells (mRNA levels) [22], gastric cancer (protein expression) [23], melanoma (protein expression) [24], lung cancer and prostate cancer (mRNA expression) [13]. Aberrant gene and protein expression of Wnt5a in PCa and possible underlying molecular mechanisms have been described in previous reports [13], [25], [26], [27]. In a recent study, based on the Affymetrix studies of normal prostate epithelial and cancer cell lines, Wang et al showed that increased transcription of the Wnt5a gene in PCa was due to hypomethylation; suggesting that epigenetic regulation of Wnt5a expression may be of importance in PCa progression [28]. Any conclusion made from data from an Affymetrix analysis without a simultaneous analysis of Wnt5a protein expression is dangerous since the Wnt5a mRNA has a long untranscribed 3′-region open for translational regulation. Data supporting such a translational regulation of Wnt5a protein expression has previously been reported [19], [29].

Recent studies have shown increased Wnt5a and protein levels in PCa compared to benign tissue [25], [26]. Yamamoto et al demonstrated *in vitro* that knockdown of Wnt5a reduced the invasive properties of DU145, and over-expression of Wnt5a stimulated invasion of PC3 cells [25]. In contrast, Wang Q and co-workers demonstrated that recombinant Wnt5a did not induce an increased motility in the same PC3 cells [26]. In addition, it has been shown by immunohistochemistry (IHC) that Wnt5a expression correlated with Gleason score ≥8 in 24 patients from a cohort of 98 PCa patients that had undergone radical prostatectomy. This could indicate that Wnt5a promotes aggressiveness, since patients with low Wnt5a levels had a better relapse-free survival compared to patients with high Wnt5a levels [25].

Conflicting reports on the role of Wnt5a in PCa progression and sparse information about Wnt5a expression in relation to clinical outcome, urged us to investigate protein expression of Wnt5a in a large population-based cohort and its possible role to predict outcome after surgery for localized and predominantly low-grade (91%) PCa. This investigation was complemented with *in vitro* experiments to explore possible reasons for the ability of Wnt5a to act as a predictive biomarker in this patient category. In the present study we confirmed that Wnt5a protein levels were upregulated in PCa compared to benign tissue but we found that increased Wnt5a protein expression had a positive effect on outcome in PCa patients, as patients with high Wnt5a protein levels had a better outcome compared to patients with low Wnt5a levels after radical prostatectomy. In good agreement, we also found that this ability of Wnt5a to positively affect outcome in PCa patients might be due to its ability to inhibit invasion of PCa cells without initially affecting their proliferation *in vitro*. Addition of Foxy5 (a Wnt5a mimicking peptide) also decreased invasion but not proliferation of these cells.

## Results

### Immunohistochemical evaluation of Wnt5a, AR, Ki-67 and VEGF

**Figure 1:**
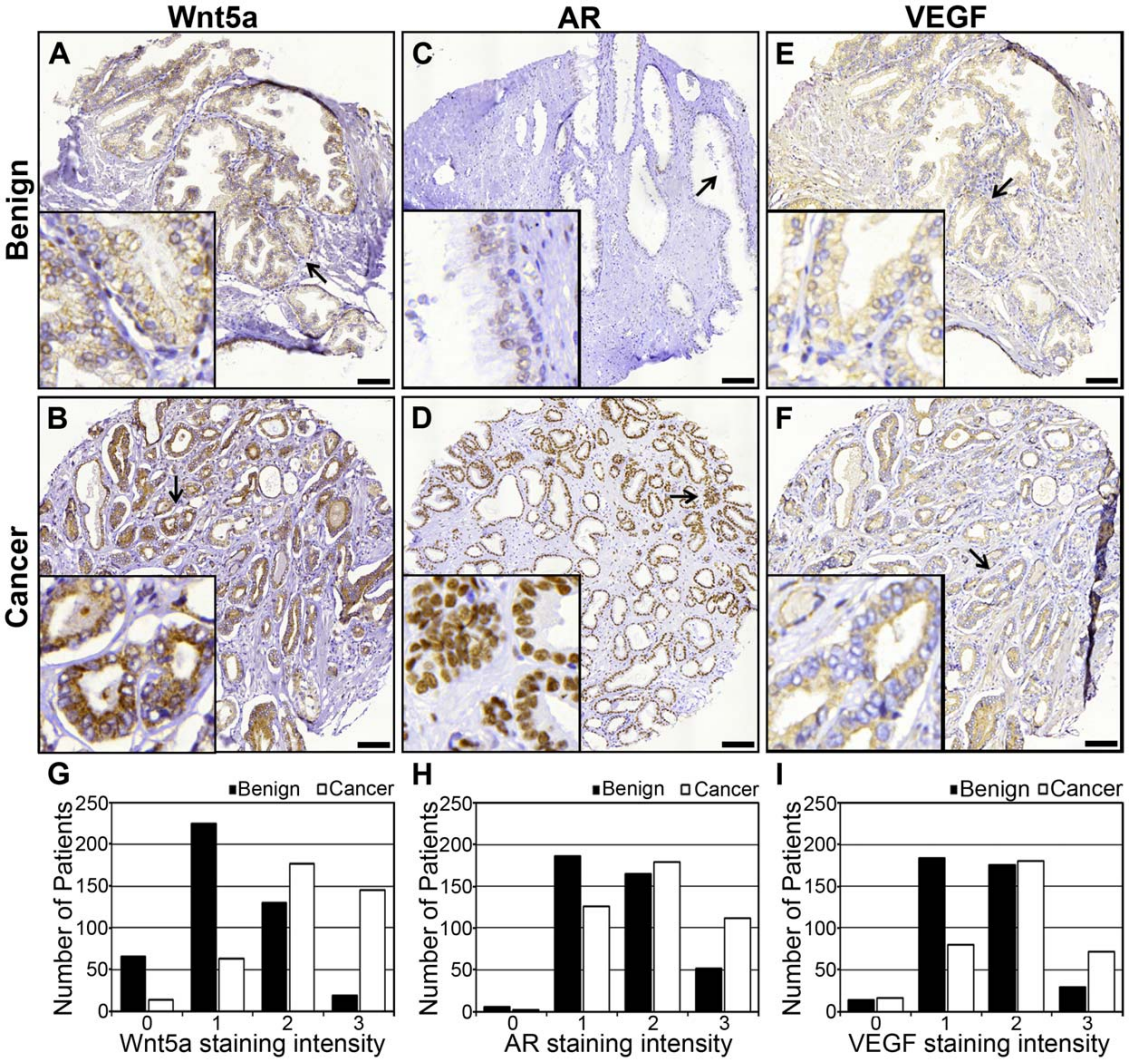
Immunohistochemical expression of Wnt5a, AR and VEGF in tissue microarray cores of primary tumors and benign specimens obtained after radical prostatectomy. **A & B**) The panels show representative Wnt5a immunostainings in benign and cancer tissue areas from the same patient **C & D**) The panels show representative nuclear AR immunostainings in benign and cancer tissue areas **E & F**) The panels outline VEGF immunostaining in benign and cancer tissue areas from the same patient. All inserts in the panels depict magnification (40×) images of the area indicated by the arrow in the larger image seen at 15× magnification. **G, H & I**) The panels outline graphical illustrations of Wnt5a, AR and VEGF protein expressions in benign and cancer samples in PCa patients. The bar in each panel outlines 100 µm.

A tissue microarray (TMA) construct with duplicate cores of both benign and malignant tissues from 503 patients (patients' characteristics in **Table 1**) that had undergone radical prostatectomy, was immunostained for Wnt5a, AR, Ki-67 and VEGF (**Fig. 1A–F**). Wnt5a protein expression was detected in the cytoplasmic compartment of epithelial cells and occasionally in stromal cells of both cancer and benign tissue specimens. Cancer tissues from most patients (82%) showed a homogenous strong cytoplasmic immunostaining, whereas a majority of benign tissues (65%) showed weak immunoreaction supporting that an up-regulation of Wnt5a protein occurs in cancer tissue. Results from manual scoring of cytoplasmic staining intensities in malignant and benign epithelial cells are illustrated in **Fig. 1G–I**. The difference between Wnt5a staining intensities in cancer and benign samples was found to be significant (p<0.0001) when paired Wilcoxon rank sum test was performed. In nearly 80% of the patients we found strong Wnt5a staining intensity (arbitrary unit 2 or 3) in cancer cores, whereas only 35% patients displayed strong staining in benign tissue samples. Further details on the scoring data from Wnt5a, AR, Ki-67 and VEGF stained cores are given in **Table 2**.

**Table 1:** Summary of patient characteristics (n = 503).

Characteristic	Median (IQR) or Frequency (%)
Age at diagnosis (years)	63.13 (59.33, 66.18)
Preoperative PSA (ng/ml)	7.2 (5.03, 11.07)
**Clinical Stage**	
T1	181
T2	233
T3	9
**Pathological Gleason Score**	
≤3+4	423 (84.1%)
≥4+3	51 ((10.1%)
Extracapsular extension	250 (49.7%)
Seminal vesicle invasion	55 (10.9%)
Positive surgical margins	259 (51.5%)
Lymph node involvement (LNI)*	3 (2%)

Abbreviation: IQR, Interquartile range.

*Information about LNI was available only for 153 patients.

**Table 2:** Scoring data from Wnt5a, AR, VEGF and Ki-67 immunostained cores from benign and cancer tissues in duplicates mounted in a TMA.

	Wnt5a		AR		VEGF		Ki-67	
Score	Benign	Cancer	Benign	Cancer	Benign	Cancer	Benign	Cancer
0	60 (15)	14 (4)	6 (1.5)	2 (0.5)	14 (3)	16 (5)	55 (14.2)	21 (5.3)
1	205 (50)	53 (14)	186 (45.6)	126 (30.1)	184 (46)	80 (23)	323 (83.2)	341 (85.5)
2	123 (30)	162 (44)	165 (40.4)	179 (42.7)	175 (44)	180 (52)	9 (2.3)	33 (8.3)
3	19 (5)	141 (38)	51 (12.5)	112 (26.7)	29 (7)	72 (21)	1 (0.2)	4 (1)
**Total**	**407 (100)**	**370 (100)**	**408 (100)**	**419 (100)**	**402 (100)**	**348 (100)**	**388 (100)**	**431 (100)**
Missing	57	94	56	45	62	116	76	65
Total	464	464	464	464	464	464	464	464
**p-value**		**<0.0001**		**<0.0001**		**<0.0001**		**<0.0001**

Scoring is based on arbitrary units with 0 representing no staining, 1 as weak staining, 2 as moderate staining and 3 as strong staining. For Ki-67 the percentage of nuclear positivity was scored as 0 (0–1% positive nuclei), 1 (1–3% positive nuclei), 2 (4–10% positive nuclei) and 3 (11–20% positive nuclei). The p values at the bottom row of the table indicate statistically significant differences between benign and cancer samples from same patient when Wilcoxon rank sum tests were performed. The values in the brackets represent number of patients (%) based on the highest score from each individual duplicate. Patients who underwent radiation therapy and/or hormonal therapy before radical prostatectomy were excluded from the IHC analysis.

Androgen receptor staining was predominantly nuclear as expected and in general more intense in cancer compared to benign tissue specimens as detailed in **Table 2**. Seventy per cent of tumor cores were intensely stained compared to 53% of benign cores.

Nuclear Ki-67 expression was used as a proliferation marker (**Figure S1A,B,C,D**). There were significant differences in Ki-67 staining between cancer and benign cores, as 14% of the benign cores were negative for Ki-67, whereas only 5% of the cancers cores were Ki-67 negative. Regarding positive Ki-67 nuclear staining, nearly 9% of the cancer cores had a staining score more than 2, whereas the corresponding number for the benign cores was only 2.5% (**Table 2**).

VEGF expression, as a surrogate marker for angiogenesis, was observed in the cytoplasm of both malignant and benign epithelial cells, with cancer areas showing higher staining compared to benign. More than 73% of the cancer cores showed strong VEGF immunostaining, whereas only 51% of the benign cores showed strong immunoreaction (**Table 2**).

The difference between AR, Ki-67 and VEGF staining intensities in cancer versus benign cores was statistically significant (p<0.0001) when Wilcoxon rank sum test was performed (**Table 2**).

### Correlation of Wnt5a tissue expression with AR, Ki-67 and VEGF

In the present cohort Wnt5a expression showed a positive and statistically significant correlation with VEGF expression (Spearman's rho (ρ) = 0.396, p<0.0001), weak but still statistically significant correlations with AR expression (ρ = 0.159, p = 0.007) and Ki-67 expression (ρ = 0.233, p<0.0001) (**Table 3**). Most of the patients (220/365, 60%) with strong Wnt5a immunostaining in cancer tissues also exhibited intense AR staining (**Table 4**). A similar trend was observed when Wnt5a and VEGF were compared; 65% (219/339) of the cancer cores exhibited strong staining for both Wnt5a and VEGF. We found no differences in Wnt5a immunostaining intensity when we compared groups of patients with different Gleason scores. Among patients with pathological Gleason score up to 3+4 (“low grade”), 81% had elevated Wnt5a protein expression compared to 86% of the patients with higher Gleason score (data not shown). Similarly, no correlation was observed between Wnt5a staining and pathological T stage, clinical T stage, surgical margin status or seminal vesicle invasion (data not shown).

**Table 3:** Spearman's correlation coefficients (ρ) when Wnt5a protein expression was analyzed for possible correlation with other tissue biomarkers in the cancer cores from 464 PCa patients.

		Ki-67	AR	VEGF
Wnt5a	ρ	0.212**	0.142**	0.395**
	p-value	<0.0001	0.007	<0.0001

**Correlation is significant at the 0.01 level (2-tailed).

**Table 4:** Multivariate analysis of factors influencing biochemical relapse-free survival.

Factors	Groups	n (%)	Hazard ratio (95% CI)	χ^2^	p - value
Wnt5a Staining	High	321 (80.7)	1 (Reference)	10.863	0.001
	Low	77 (19.3)	2.204 (1.361–3.570)		
Wnt5a & Ki67 staining	Wnt5a high Ki67 low	255 (73.1)	1 (Reference)	36.638	
	Wnt5a low Ki67 low	59 (16.9)	2.335 (1.344–4.054)		0.003
	Wnt5a low Ki67 high	3 (0.6)	14.501 (4.412–47.658)		<0.0001
	Wnt5a high Ki67 high	32 (9.2)	2.215 (1.128–4.351)		0.021
Wnt5a & AR staining	Wnt5a high AR low	81 (22.2)	1 (Reference)	19.769	
	Wnt5a low AR low	28 (7.7)	3.044 (1.067–8.685)		0.037
	Wnt5a low AR high	36 (9.9)	6.060 (2.489–14.756)		<0.0001
	Wnt5a high AR high	220 (60.3)	2.503 (1.129–5.546)		0.024
Wnt5a & VEGF staining	Wnt5a high VEGF low	63 (18.6)	1 (Reference)	13.955	
	Wnt5a low VEGF low	29 (8.6)	2.843 (1.121–7.211)		0.028
	Wnt5a low VEGF high	28 (8.3)	3.501 (1.407–8.712)		0.007
	Wnt5a high VEGF high	219 (64.6)	1.323 (0.617–2.836)		0.472
Path. Gleason Score	≤3+4	398 (90.5)	1 (Reference)	9.302	0.002
	≥4+3	42 (9.5)	2.247 (1.317–3.835)		
Path. T Stage	T2	226 (50.4)	1 (Reference)	22.05	<0.0001
	T3	222 (49.6)	2.655 (1.738–4.056)		

n = Frequency; CI = Confidence Interval; χ^2^ = Chi-Square; Path. = Pathological.

### Wnt5a protein expression and prediction of clinical outcome

**Figure 2:**
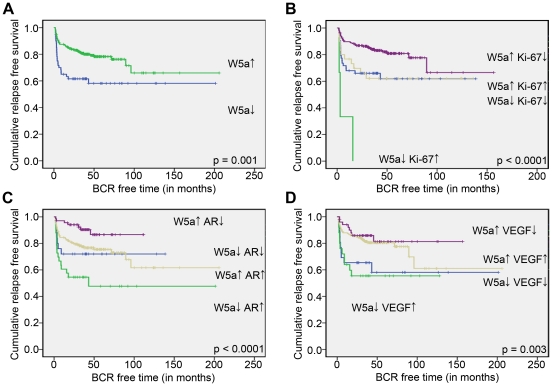
Analysis of how Wnt5a protein expression alone or in combination with other biomarkers affects the clinical outcome of PCa patients. All cancer cases were separated into 2 groups based on the staining intensities of Wnt5a, Ki-67, AR and VEGF. The low groups included tumors with scores 0 or 1 and the high groups included tumors with scores 2 or 3. **A**) The panel shows survival curves plotted between high or low Wnt5a protein expression and BCR free time. **B**) The panel shows survival curves plotted between high or low Wnt5a and high and low Ki-67 protein expressions. Consequently, the tumors were divided into the following 4 groups; Wnt5a low & Ki-67 low, Wnt5a low & Ki-67 high, Wnt5a high & Ki-67 low and Wnt5a high & Ki-67 high. **C** The panel shows survival curves plotted between high or low Wnt5a and high and low AR protein expressions. Consequently, the tumors were divided into the following 4 groups; Wnt5a low & AR low, Wnt5a low & AR high, Wnt5a high & AR low and Wnt5a high & AR high. **D**) The panel shows survival curves plotted between high or low Wnt5a and high and low VEGF protein expressions. Consequently, the tumors were divided into the following 4 groups; Wnt5a low & VEGF low, Wnt5a low & VEGF high, Wnt5a high & VEGF low and Wnt5a high & VEGF high. In all panels high expression of a protein is indicated by ↑ whereas ↓ indicates low expression. Each step in the curves represent relapse in PCa. The given p-values at the bottom right hand side of the panels indicate significant differences in outcome between the most favorable group and the least favorable group (see **Table 4** for more detailed information).

Next, we evaluated if Wnt5a protein expression in cancer tissues analyzed after radical prostatectomy for localized PCa could predict clinical outcome as measured by time to biochemical recurrence (BCR), using PSA >0.2 ng/mL in blood samples with a confirmatory value as a surrogate marker. Wnt5a protein expression as illustrated by IHC was significantly higher in cancer areas compared to benign areas (**Fig. 1** **,** **Table 2**). Interestingly, when Kaplan-Meier curve was plotted between Wnt5a protein expression and BCR free time, a favourable outcome (p = 0.001) was evident for patients with a high Wnt5a protein expression compared to patients with low Wnt5a protein expression (**Fig. 2A**). As expected, low expression of AR (**Figure S2C**) and of Ki-67 (**Figure S2B**) was associated with favorable outcome whereas VEFG expression was not significantly associated with BCR free time (**Figure S2D**).

Further, we examined if Wnt5a protein expression also could predict outcome when combined with any of the other tissue biomarkers. The best prediction model was obtained when Wnt5a protein expression was combined with either AR or Ki-67 expression (**Fig. 2B, C**), as patients with high Wnt5a and low AR or low Ki-67 expression showed better relapse free survival (p<0.0001), whereas patients with low Wnt5a expression and high AR or high Ki-67 expression had the worst outcome after surgery. Patients with high Wnt5a and low VEGF expression had better outcome compared to other groups (p = 0.003) or each marker alone. However, the combination of high Wnt5a and low VEGF was inferior to when Wnt5a was analyzed in combination with AR or Ki-67 indicating that VEGF in not as strong as AR or Ki-67 to predict outcome in combination with Wnt5a in the present context (**Fig. 2D**). Cox regressional analysis was used for multivariate analyses and revealed that Wnt5a expression, Gleason score and pathological T stage were independent factors influencing relapse free survival in PCa (**Table 4**).

### Wnt5a protein expression and its effects on invasion and proliferation of PCa cell lines

**Figure 3:**
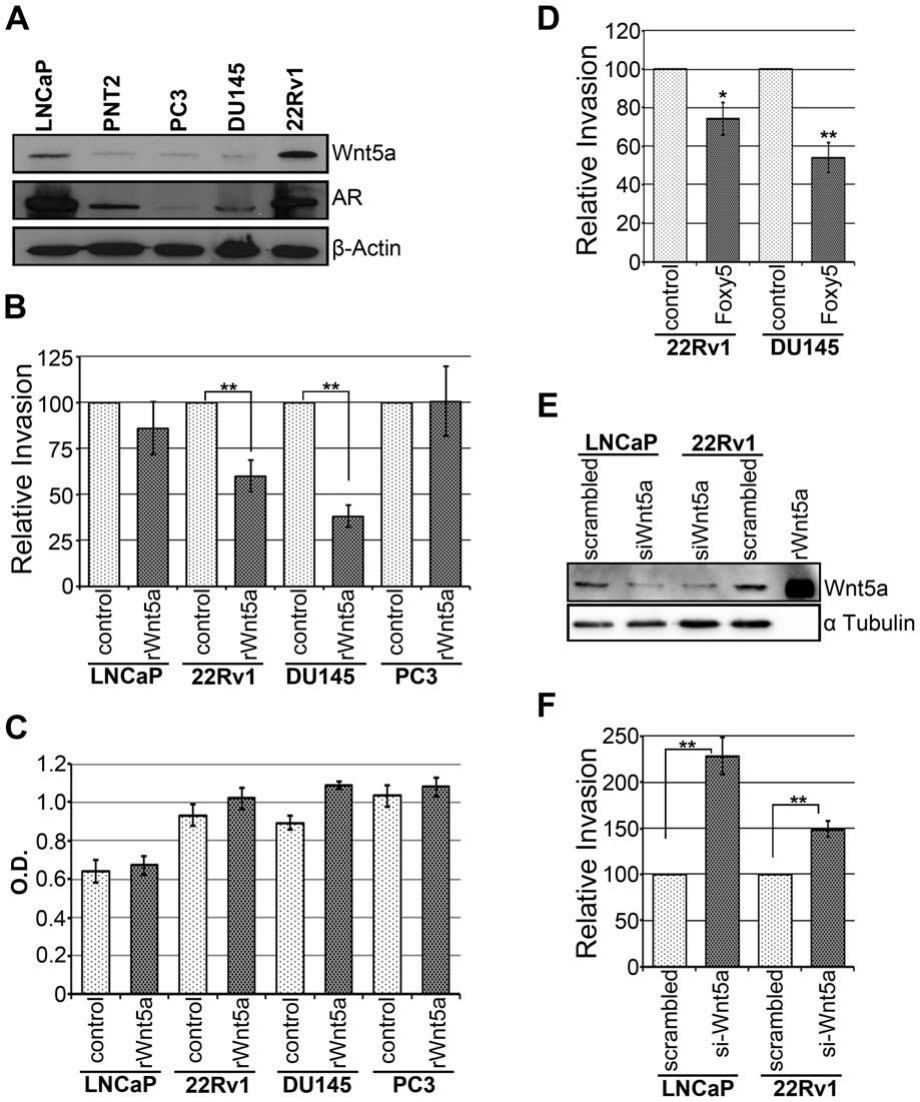
Analysis of Wnt5a protein expression in different prostate cell lines and its effect on PCa cell invasiveness and proliferation. **A**) This panel shows the endogenous Wnt5a and AR expression in four indicated PCa cell lines (LNCaP, 22Rv1, DU145 and PC3 cells) and in one immortalized human prostate epithelial cell line (PNT2 cells). Wnt5a protein band was identified by running rWnt5a in parallel on the same gel. The blots were reprobed for β-actin as loading control. The presented blots are representative of 4 separate experiments. **B**) The panel outlines the relative invasion of LNCaP, 22Rv1, DU145 and PC3 cell lines after 24 h in the absence or presence of rWnt5a (0.4 µg/ml) in the assay described in the Materials and Methods section. The results are given as means ± SEM from 5 separate experiments. The differences in invasion between cells treated with vehicle alone or with rWnt5a were evaluated for statistical significance (p = 0.0001 for 22Rv1 and p<0.0001 for DU145). **C**) The panel outlines the proliferation of LNCaP, 22Rv1, DU145 and PC3 cell lines after 24 h in the absence or presence of rWnt5a (0.4 µg/ml). The results are given as means ± SEM from 5 separate experiments. There were no significant differences in proliferation between control and rWnt5a treated cells. **D**) The panel represents the relative invasion of 22Rv1 and DU145 cells after 24 h in the absence or presence of Foxy5 (100 µM) using the same assay as in panel B. The differences in invasion between cells treated with vehicle alone or with Foxy5 were evaluated for statistical significance (p = 0.01 for 22Rv1 and p = 0.0003 for DU145). **E**) The panel depicts the effects of siRNA knockdown of Wnt5a in LNCaP and 22Rv1. The blots were reprobed for α-tubulin as loading control. The presented blots are representative of 4 separate experiments. **F**) The panel outlines the relative invasion of LNCaP and 22Rv1 cells after treatment with Wnt5a si-RNA (Wnt5a knockdown) or scrambled siRNA (control), in the same assay previously used in panel B. The results are given as means ± SEM from 5 separate experiments. The difference in invasion between scrambled and si-Wnt5a knocked down cells were statistically significant for both cell lines (p<0.0001).

Our data show that patients with high Wnt5a protein expression have more favorable outcome compared to patients with low Wnt5a protein expression. To better understand this finding Wnt5a protein expression in one SV40 immortalized normal human prostate epithelial cell line (PNT2) and four different PCa cell lines were examined. PNT2 cells had a low expression of endogenous Wnt5a protein whereas there was considerable Wnt5a expression in the PCa cell lines LNCaP and 22Rv1 (**Fig. 3A**). These data are in good agreement with our findings of Wnt5a protein expression in the presently analyzed cohort of PCa and a recent study also performed on normal prostate epithelial and PCa cell lines [26]. However, analyses of the two more aggressive PCa cell lines (PC3 and DU145) revealed very low expression of Wnt5a protein, comparable to that of the normal PNT2 prostate epithelial cell line (**Fig. 3A**). Interestingly enough, the Wnt5a protein levels matched with the AR protein expressions in these cell lines (**Fig. 3A**).

We used the four cancer cell lines (LNCaP, 22Rv1, PC3 and DU145) for our subsequent invasion experiments. Addition of recombinant Wnt5a (rWnt5a) decreased the invasive behavior of both 22Rv1 and DU145 cancer cells (**Fig. 3B**). Neither the LNCaP nor the PC3 cells did respond to rWnt5a with a change in their invasive behavior. The result with the PC3 cells is in accordance to a recently published report by Wang et al [26], in which PC3 cells did not respond to addition of rWnt5a in a migration wound scratch assay. LNCaP cells are known to have a very low invasion activity, and this might explain why these cells did not respond when rWnt5a was added. However, when Wnt5a expression in LNCaP cells was knocked down using si-RNAs (**Fig. 3E**), there was a significant increase in the invasive behavior of LNCaP cells (**Fig. 3F**). In addition, Wnt5a knockdown by si-RNA in 22Rv1 cancer cells also resulted in increased invasion of these cells (**Fig. 3E–F** **and Figure S4**).

To find out whether the decrease in invasion of 22Rv1 and DU145 cell lines with the addition of rWnt5a was due to decrease in proliferation of these cell lines, we investigated the proliferation rate in PCa cell lines. Addition of rWnt5a did not have any significant effect on proliferation in these cell lines during the 24 hours used for the invasion assay (**Fig. 3C**).

Since rWnt5a decreased the invasion of 22Rv1 and DU145 cells, invasion assay was also performed in these cell lines using Foxy5 which is a hexapeptide derived from the amino acid sequence of Wnt5a protein and previously shown to inhibit motility of breast cancer cells like rWnt5a [30]. Foxy5 indeed repressed invasive capabilities of these two PCa cell lines (**Fig. 3D**), and this decrease in invasion was not caused by decreased proliferation as Foxy5 did not affect the proliferation status in these cell lines (BrdU assay, data not shown).

## Discussion

To our knowledge, this far only one study with a limited number of patients has demonstrated a role of Wnt5a protein to predict clinical outcome in PCa [25]. This urged us to perform a study on Wnt5a protein expression in a larger cohort of well-defined PCa patients with localized and predominantly low-grade disease and relate the results with the expression of other known tissue biomarkers and most importantly with BCR. The present study involved a consecutive series of 503 PCa patients that had undergone radical prostatectomy during 1988–2003 at Skåne University Hospital, Malmö, Sweden with a mean follow-up time of 41.6 month (range 1.51–205.90). This patient cohort is large, population based, and the patients are well characterized (**Table 1**). In the TMA slides benign and malignant tissues from the same patient are present in duplicates. Based on Gleason grades patient material was further characterized into low-grade cancers (Gleason score up to 3+4) and high-grade cancers (Gleason 4+3 or higher). Almost 89% of the patients were classified as low-grade cancers, which is to be expected in a group of patients with localized PCa suitable for radical prostatectomy. As a control of our clinical material, we ascertained that there was a statistically significant difference in clinical outcome between patients with low-grade and high-grade cancer using Kaplan-Meier analyses of BCR-free survival (**Figure S2A**). Further control of the clinical material also revealed that the same was true when proliferation was studied by Ki-67 expression, a validated tissue biomarker in PCa [31]. Patients with high Ki-67 expression had reduced relapse free survival time when compared with patients with a low number of Ki-67 expressing tumor cells (**Figure S2B**).

In the present TMA study we used a well characterized in-house antibody specific for Wnt5a as previously described in breast cancer studies [20]. Here, we also performed competition with rWnt5a to confirm the specificity of the antibody on prostatic tissue sections (**Figure S3**). The staining intensity decreased from antibody alone to when antibody and rWnt5a were used and already at a molar ratio of 1∶10 we found a clear reduction of the immunostaining. In addition, we carried out immunocytochemistry (**Supplementary Materials and Methods S1**) of Wnt5a in prostate cancer cell lines (LNCaP, 22Rv1 and DU145) after pretreatment with either scrambled or Wnt5a si-RNA (**Figure S4A,B,C,D,E**). First, we observed cytosolic staining of Wnt5a similar to that observed in the prostate cancer tissue and secondly, the intensity of Wnt5a immunostaining decreased significantly in the Wnt5a si-RNA treated cells compared with those treated with scrambled si-RNA. Treatment with Wnt5a siRNA decreased the degree of Wnt5a immunostaining to a level similar to that seen in the Western blots (**Fig. 3E**). Analysis of our TMA clearly show that Wnt5a protein expression was increased in localized PCa when compared to benign tissue from the same patients, an effect that exhibited a strong statistical significance (p<0.0001; **Fig. 1 A, B & G** **,** **Table 2**). These results are in good agreement with the recent findings obtained from a smaller cohort [25]. The clinical conclusion that Wnt5a protein expression is increased in localized PCa tissue compared with normal/benign tissue is also supported by our analysis of different human prostate cell lines. We clearly observed that the PNT2 cell line, an SV40 immortalized cell line derived from normal human prostate epithelium express very low levels of endogenous Wnt5a protein, whereas the expression of Wnt5a protein was high in the PCa cell lines LNCaP and 22Rv1. The more aggressive cell lines, PC3 and DU145, had a very low Wnt5a protein expression. This is in line with the less favorable outcome observed in Wnt5 low tumors. However, in the TMA material, Wnt5a was not downregulated in the high-grade (Gleason score >4+3) PCa cases. If these seemingly contradicting results indicate a grade-unrelated function of Wnt5a or only reflect the individual characteristics of the two tumors from which the cell lines were derived is hard to say. As an alternative explanation, the number of high-grade PCa in the present cohort (n = 41) might be too small to detect a grade-related Wnt5a down-regulation.

We also found increased expression of AR, Ki-67 and VEGF proteins in localized PCa tissue compared to benign tissue (**Fig. 1 C–F, H–I** **,** **Table 2**). To obtain a first insight into possible mechanisms for how Wnt5a functions in PCa, we performed statistical analyses of potential correlations between Wnt5a protein expression and that of AR, Ki-67 and VEGF, all three well-known to be upregulated in progressive PCa. Wnt5a significantly correlated with VEGF, a marker for angiogenesis, indicating that Wnt5a might be related to tumor growth (**Table 3**). In this regard our data is somewhat different from those reported from analyses of non-small cell lung cancer where Wnt5a did not correlate with VEGF expression in the cancer tissue but with VEGF in the surrounding stromal tissue [32]. Furthermore, Wnt5a expression in PCa tissue in our study weakly but significantly associated with AR expression (**Table 3**). Protein expression analysis by western blot indicated that Wnt5a levels and AR expression in one immortalized prostate epithelial cell line and 4 different PCa cell lines matched with each other, indicating a possible correlation between Wnt5a and AR in PCa (**Fig. 3A**). Despite these data, it has been recently shown that Wnt5a inhibits AR transcriptional activity in 22Rv1 cells when these cells were transfected with a Wnt5a plasmid [33]. Finally, Wnt5a protein expression was weakly but significantly associated with Ki-67 expression (**Table 3**). This result is in accordance with the report on Non-small-cell lung cancer, where intratumoral Wnt5a expression significantly correlated with Ki-67 proliferation index [32], but in contrast to the study on hepatocellular carcinoma where Wnt5a has a tumor suppressing effect and loss of Wnt5a has a strong correlation with high Ki-67 proliferation index [34]. Taken together these data indicate that the role of Wnt5a signaling in the regulation of tumor cell proliferation is uncertain.

In the present investigation we did not find a correlation between Wnt5a protein expression and the Gleason score, although the latter may be the best available prognostic indicator of outcome in PCa [35]. However, Gleason scoring has its limitations due to interobserver variability among pathologists and hence there is a need for complementary markers. To determine whether or not Wnt5a protein expression can be used to predict outcome (relapse-free survival) after surgery in patients with localized PCa in this population-based cohort, Kaplan-Meier curves were plotted between Wnt5a protein expression and BCR free time (**Fig. 2A**). Interestingly, patients with high Wnt5a protein expression had a statistically significant more favorable outcome compared to patients with low Wnt5a protein expression indicating that the Wnt5a protein has a tumor suppressive function in the context of localized PCa. In majority of cases, Wnt5a signaling has opposite effects than Wnt/β-catenin signaling, for example in malignant melanoma [36]. Although a different and more advanced PCa patient material was used by Chen and co-workers, their finding that Wnt1 and β-catenin expression can serve as markers for PCa progression [12] is compatible with our data that Wnt5a predicts a more favorable outcome in PCa patients.

Combining Wnt5a protein expression with other well-known PCa markers could further improve the predictive power of Wnt5a as previously mentioned. The hypothesis that Wnt5a has a tumor suppressive function was further supported by our invasion data in three of four PCa cell lines investigated. Addition of rWnt5a led to decrease in invasion in 22Rv1 and DU145 cells. It was not surprising that LNCaP cells, known to have a very low invasive behavior, did not exhibit a detectable further reduction in its invasive behavior in response to rWnt5a. Upon further investigation we found that the rWnt5a effect in 22Rv1 and DU145 cells is specific on the invasion of these cells and not because of its toxicity to these cells or has any adverse effect on proliferation of these cell lines. In both cell lines rWnt5a triggered a prompt and transient rise in the cytosolic free calcium level (**Supplementary Materials and Methods S1; Figure S5A,B**), indicative of a ligand-receptor interaction. If there would have been a toxic effect of rWnt5a one would have anticipated a slow increase that then remained elevated, but this was not the case. Furthermore, if the effect of rWnt5a would be toxic one would also have anticipated a reduction of BrdU positive cells, which we did not see. Addition of rWnt5a did not have a significant effect on proliferation of these cells. However, Wnt5a knockdown experiments were performed on LNCaP cells, as well as on 22Rv1 cells, Wnt5a siRNAs increased the invasive activity of LNCaP and 22Rv1 cells; indicating that for PCa cells to invade, Wnt5a must be actively silenced. Like rWnt5a, Foxy5 (a Wnt5a-derived hexapeptide) also affected invasion in 22Rv1 and DU145 cells without having an effect on proliferation of these cell lines. These results are in accordance with an earlier report published from our group on breast cancer metastasis where neither rWNt5a nor Foxy5 affected proliferation or apoptosis but inhibited migration and invasion in 4T1 breast cancer cells [37].

It has recently been suggested that Wnt5a promotes aggressiveness of PCa and patients with low/negative Wnt5a expression have better relapse free survival after radical prostatectomy [25]. These results are quite in contrast to our findings. Their contrasting results can be attributed to less patient samples and the fact that in their material 24.5% (24 out of 98 patients) of the tumors had a Gleason score of 8 or higher, whereas in our study only 11% of the tumors had such a high Gleason score. Furthermore, different Wnt5a antibodies were used in the two studies. Our Wnt5a antibody has been evaluated by peptide blocking experiments during IHC [20], loss of Wnt5a following siRNA knockdown and Wnt5a overexpression. However, it cannot be excluded that Wnt5a exerts different effects on tumor progression in different stages of the disease. Our different results from the *in vitro* invasion assay can possibly be explained by the fact that we have used a defined concentration of rWnt5a and the other group used cells transfected to over-express Wnt5a without any control of the actual stimulating concentration of Wnt5a.

There are studies within the scientific community on the possible role of Wnt5a in suppressing or promoting tumor progression. It must be pointed out that an upregulation of Wnt5a mRNA in a specific cancer type does not alone indicate a tumor promoting function, since this might very well go hand in hand with a reduced Wnt5a protein level. Even if this is taken into account it appears as if Wnt5a has different functions in different types of tumors [16]. In conclusion, our study indicates that although Wnt5a protein expression is elevated in PCa, its expression in PCa cells is associated with a more favorable outcome for patients with localized disease. One important mechanism for such an effect of Wnt5a in PCa progression is the present demonstration that Wnt5a can impair the invasive behavior of PCa cells *in vitro*. Taken together, our results suggest a novel therapeutic approach for patients with localized PCa by targeting Wnt5a to impair progression of PCa in these patients.

## Materials and Methods

### Ethics statement

This study was performed after approval from Regional Ethical Review Board in Lund. Archival tissue specimens from 503 patients operated on between 1988 and 2003 were used in the present study. Since some of the samples were old, and were from different parts of the region, it was not possible to obtain written consent from each patient. Detailed information describing the study and TMA construction was published in 2004 in a daily newspaper and patients had a free choice of written or verbal informed consent as they were offered to contact us by mail or by phone if they had any objections. None of the 503 patients declined. This procedure was done strictly following guidelines from Regional Ethical Review Board in Lund who approved the procedure.

### Patients and tissue microarray (TMA) construct

A TMA was constructed from a population-based cohort of 503 PCa patients who underwent radical prostatectomy between 1988 and 2003 at the Department of Urology, Skåne University Hospital, Malmö, Sweden as previously described [38]. From each patient, benign and malignant cores in duplicate were mounted in a total of 17 paraffin blocks. Consecutive sections were used for IHC. A senior National Board certified pathologist (LH) examined hematoxylin & eosin stained tissues for Gleason grade and for the presence of prostatic intraepithelial neoplasia. The clinical and pathological characteristics of the PCa patients were obtained from reading the patient charts in detail (DU and AB) and are shown in **Table 1**. The mean follow-up time was 41.6 months (range 1.51–205.90). BCR was defined as a blood PSA level of at least 0.2 ng/ml with a subsequent confirmatory value.

### Source of antibodies

The following antibodies were used for immunostainings: Wnt5a (rabbit polyclonal): antibody was developed in our laboratory against a Wnt5a sequence with 100% homology between human and mouse [20]; androgen receptor (AR) (code AR 441, mouse monoclonal, Thermo Fisher Scientific Inc., Freemont, CA), Ki-67 (mouse monoclonal, MIB-1 code M7240, Dako Denmark A/S, Glostrup, Denmark); VEGF A-20 (rabbit polyclonal, code sc-152, Santa Cruz Biotechnology, Inc., Santa Cruz, CA); β-actin (mouse monoclonal, code C4, MP Biomedicals, Solon, OH), α Tubulin (mouse monoclonal, sc-32293, Santa Cruz Biotechnology).

### Immunohistochemistry (IHC)

Consecutive sections of 4 µm thicknesses were mounted on Superfrost Plus (Menzel Gläser, Braunschweig, Germany) glass slides and de-paraffinized with xylene and rehydrated in decreasing concentrations of ethanol solutions. For antigen retrieval TMA slides were heated in PT Link (Dako) from 65°C to 98°C for 40 min and then processed for immunohistochemical staining for Wnt5a (final dilution 1∶100), AR (1∶100), Ki67 (1∶100) and VEGF (1∶100) using EnVision™ Flex, High pH reagent (code K8010, Dako) in Autostainer Plus according to the manufacturer's protocol (Dako). Immunostaining of Wnt5a, Ki-67, AR and VEGF were scored independently by pathologists LH, AE and RE. Overall, scoring pattern matched in nearly 80% of cases in staining intensities as well as percentage of positive cells. Remaining 20% cases where there was a disagreement over scoring were re-examined together and were scored after coming to a conclusion. In general, the cores were scored 0 (no staining), 1 (weak staining), 2 (moderate staining) or 3 (strong staining) based on the staining intensities and/or percentage of positive cells. Wnt5a and VEGF slides were scored based on the cytoplasmic staining whereas nuclear staining was evaluated for AR staining. Ki-67 slides were scored as 0 (0–1%), 1 (1–3%), 2 (4–10%) and 3 (11–20%) based on nuclear fraction positivity. While performing statistics protein expression scores were separated into two groups based on their staining intensities; scores 0 & 1 are grouped as weak/low and strong/high group contains scores of 2 & 3.

For IHC studies and correlation analyses on Wnt5a, Ki-67, AR and VEGF, patients with no Gleason score information available (29), and patients who received hormonal and/or radiation therapy (39) were excluded, leaving 464 patients for analyses. During TMA construction some cores were either lost, or were not properly placed on slides, or were damaged and were not available to score; hence immunostaining data of proteins contains missing values (**Table 2**).

We also performed competition with recombinant Wnt5a to confirm the specificity of the Wnt5a antibody. Prostate cancer cores were immunostained with either the Wnt5a antibody alone (**Figure S3A**) or with the Wnt5a antibody supplemented with recombinant Wnt5a (molar ratio 1∶1 (**Figure S3B**) and 1∶10 (**Figure S3C**)). The staining intensity decreased from antibody alone to when antibody and rWnt5a were used and already at a molar ratio of 1∶10 we found a clear reduction of the immunoreaction.

### Cell lines

Four human PCa cell lines LNCaP, 22Rv1, PC-3, and DU145 were purchased from American Type Culture Collection [ATCC] (Manassas, VA). The immortalized PNT2 normal human prostate epithelial cells (cat No. 95012613) were obtained from European Collection of Cell Cultures (ECACC), (Sigma-Aldrich, St. Louis, MO). LNCaP, 22Rv1, DU145 and PNT2 cells lines were cultured in RPMI-1640 medium, supplemented with 10% fetal bovine serum (FBS) and 1% pest (penicillin and streptomycin). PC-3 cells were grown in Hyclone Ham's F12 medium supplemented with 10% FBS and 1% pest. All *in vitro* experiments were performed when cells were ∼70% confluent. For invasion assay experiments cells were grown in serum free medium (SFM) for 24 hours. RPMI-1640 medium (R0883), FBS (F6178), penicillin-streptomycin (P0781) were purchased from Sigma-Aldrich, whereas Ham's F12 medium (SH30026.01) was obtained from Thermo-Scientific (Waltham, MA). All cell lines were regularly tested for the absence of mycoplasma infection.

### Western blot Analysis

Protein expression was examined by western blot analysis. In brief, cells were washed with PBS, trypsinized (in trypsin for 3 min), centrifuged at 1000 rpm for 4 minutes. Cells were lysed on ice in RIPA buffer (50 mM Tris–HCl pH 7.4, 150 mM NaCl, 1% Triton x-100, 1% sodium deoxycholate, 0.1% sodium dodecyl sulfate, 1 mM EDTA, 0.1 mg/mL Phenylmethylsulphonyl fluoride with the addition of Complete Mini protease inhibitor cocktail (Roche, Mannheim, Germany) for 30 min, centrifuged at 15,000 rpm for 25 min at +4°C, and protein lysates were collected as supernatants. After measuring protein concentration by Bradford assay, 100 µg of each protein sample was loaded on 10% SDS – polyacrylamide gels. Proteins were separated using gel electrophoresis and transferred to Hybond ECL nitrocellulose membranes (Amersham Pharmacia Biotech, Buckinghamshire, UK). For blocking of non-specific binding, nitrocellulose membrane was blocked in 5% dry milk for 45 min at room temperature, washed twice in buffer (0.05% Tween in PBS) for 10 min, and then incubated overnight separately with rabbit polyclonal Wnt5a antibody (1∶750 in 2.5% dry milk) or mouse monoclonal AR (1∶500 in 2.5% dry milk) at +4°C. After incubation (for 60 min at room temperature) with horseradish peroxidase – conjugated anti-rabbit secondary antibody (Amersham Life Science, Alesbury, UK) (1∶10000 in 5% dry milk) for Wnt5a and horseradish peroxidase – conjugated goat anti-mouse secondary antibody (Dako) (1∶10000 in 5% dry milk) for AR and washing away the unbound antibodies, membrane-bound antibody was detected by using Western blotting Luminol Reagent (Santa Cruz). Membranes were then stripped using stripping solution (Restore PLUS Western Blot Stripping Buffer, Thermo Scientific) and reprobed for β-actin (1∶3,000 in 2.5% dry milk) or α Tubulin(1∶1000 in 2.5% dry milk).

### Transfection with Wnt5a siRNA

Two different Silencer® Select Pre-designed (Inventoried) Wnt5a siRNAs (S1 and S2) and Silencer® Select negative control siRNA were purchased from Applied Biosystems (Ambion, CA). A cocktail of two different siRNAs (120 nM) in nuclease-free water was transfected into 1×10^5^ cells in a total volume 250 µL of serum free medium using 10 µL of Lipofectamine 2000 (Invitrogen, Carlsbad, CA). Media was changed after 5 hours of transfection. After 24 hours of transfection, media was changed to SFM, and cells were used 24 hours later for analysis of their Wnt5a protein expression and invasive capacities.

### Invasion Assay

Cell invasion capacities were measured in a standard commercial invasion assay. In this study we used BD BioCoat™ Matrigel™ Invasion Chambers (BD Biosciences, Bedford, MA) in accordance with the manufacturer's protocol. Briefly, cells were grown in SFM for 24 h, harvested using versene (Invitrogen, Carlsbad, CA), washed in PBS and resuspended at a concentration of 50,000 cells/ml in SFM. To the lower well 0.7 ml serum containing medium (10% FBS) was added. To the invasion chamber 0.5 ml (25,000 cells) of the cell suspension, containing either 0.4 µg/ml recombinant Wnt5a (rWnt5a, R&D Systems, Minneapolis, MN), or 100 µM Foxy5 (formyl-Met-Asp-Gly-Cys-Glu-Leu peptide, Pepscan Systems, Lelystad, Netherlands) or PBS (with 0.2% BSA) was added, and were incubated for 24 h at 37°C. After 24 h cells that invaded through the Matrigel were fixed in 4% paraformaldehyde and stained with 0.2% crystal violet in 20% methanol (Sigma-Aldrich, Saint Louis, MO, USA). Remains of the Matrigel were removed with a cotton stick moistened in PBS. Membranes from invasion chambers were separated and mounted on glass slide using VectaShield® mounting medium with DAPI (Vector Laboratories, Burlingame, CA). Invaded cells were counted either in an inverted microscope or in Olympus BX51 Fluorescence Microscope (Olympus optical Co. Ltd, Japan).

### Proliferation Assay

Cell proliferation assay was performed in LNCaP, 22Rv1, DU145 and PC-3 cells using Cell Proliferation BrdU kit version 13.0 (11647229001, Roche diagnostics, Mannheim, Germany) according to manufacturer's instructions. Briefly, 25000 cells with BrdU labeling solution were seeded in 96-well plate and incubated with either vehicle (0.01%BSA in PBS) or rWnt5a (0.4 µg/mL) for 24 h in 37°C incubator. After 24 h, cells were fixed for 30 min, incubated with anti-BrdU-POD for 90 min at room temperature and washed. Absorbance of the samples was measured in an ELISA reader at 370 nm (reference wavelength 492 nm) at multiple time points (e.g., 4, 8 and 12 min) after substrate solution was added. The results presented here are absorbance values after 4 minutes.

### Statistical analysis

All statistical analyses were performed using SPSS version 17.0 (SPSS, Chicago, IL) and Microsoft Excel 2010. Since patients' samples were present in duplicates, the best score of the two cores (if available) was used for statistical analyses. Patients receiving preoperative hormonal treatment or radiation therapy (n = 39), patients with no information available on Gleason score (29) and the patients where PSA levels were not completely 0 after radical prostatectomy and hence no BCR (n = 75) were excluded, leaving a total of 397 patients for survival and multivariate statistical analyses. For statistical analyses patient material was divided into two groups based on Gleason scoring; patients with Gleason score 5 to 7 (with 3+4 cases only) were grouped as “low-grade cancers”, and patients with Gleason score 7 (4+3 cases only) to 10 were put together as “high-grade cancer” group. Wilcoxon Signed Ranks test was used to examine any significant difference in Wnt5a protein expression between cancer and benign tissues. Spearman's rank-order correlation was performed to know significant correlations between Wnt5a, AR, Ki-67 and VEGF staining. Kaplan-Meier method was used to determine BCR-free survival (outcome) and Log Rank (Mantel-Cox) test was used to compare BCR free survival among different Wnt5a expression groups. For survival analysis staining intensities of different proteins were grouped into two; no/weak staining in group “1” (low) and moderate/strong staining in group “2” (high). In some analyses, expression pattern of two different proteins were grouped together, for example, while performing survival curves and Cox regressional analyses Wnt5a and AR staining intensities were grouped together, making four different groups. Patients with low Wnt5a and low AR staining constituted group 1, group 2 had patients with low Wnt5a and high AR staining, patients with high Wnt5a and low AR were kept in group 3, whereas group 4 consisted of patients with high Wnt5a and high AR staining intensities. The same criterion was applied while combining Wnt5a staining intensities with Ki-67/VEGF scorings.

## Supporting Information

Materials and Methods S1 (DOC)

Figure S1 Representatives of Ki-67 nuclear fraction immunostainings. **A**) The panel represents cancer core with no Ki-67 nuclear staining. **B**) The panel represents cancer core with 1–3% Ki-67 nuclear staining, **C**) The panel shows cancer core with 4–10% of nuclei stained positive for Ki-67 **D**) The panel shows cancer core with more than 10% of nuclei stained positive for Ki-67. All inserts in the panels depict magnification (40×) images of the area indicated by the arrow in the larger image seen at 15× magnification. The bar in each panel outlines 100 µm.
(TIF)

Figure S2 Validation of the patient material used in this study. **A**) The patient tumor material was divided into 2 groups based on their Gleason score (GS). As indicated in the panel one group had a Gleason score of ≤3+4 and the other a Gleason score of ≥4+3. Kaplan-Meier curves were then generated for each of the 2 groups with the indicated Gleason scores and their respective BCR free time. **B**) The panel shows Kaplan-Meier curves plotted between low or high Ki-67 expression and their respective BCR free time. **C**) The panel shows Kaplan-Meier curves plotted between low or high AR expression and their respective BCR free time. **D**) The panel shows Kaplan-Meier curves plotted between low or high VEGF expression and their respective BCR free time.
(TIF)

Figure S3 Validation of Wnt5a antibody specificity by blocking with rWnt5a. **A** shows a prostate cancer core section immunostained with anti-Wnt5a IgGs alone. **B & C**) **A**djacent tissue sections immunostained using the same Wnt5a antibody after pre-incubated with rWnt5a at a molar ratio of 1∶1 or 1∶10, respectively. Each bar outlines 100 µm.
(TIF)

Figure S4 Immunocytochemistry of prostate cancer cell lines after Wnt5a knockdown using si-RNA, immunostained with Wnt5a antibody. **A**) Wnt5a staining in LNCaP cells transfected with scramble RNA. **B**) Decreased intensity of Wnt5a staining in LNCaP cells transfected with si-Wnt5a. **C**) Wnt5a staining of 22Rv1 cells transfected with scramble RNA. **D**) Decreased Wnt5a staining in 22Rv1 cells transfected with si-Wnt5a. **E**) Weak Wnt5a immunostaining in DU145 cells.
(TIF)

Figure S5 Measurement of intracellular Ca^2+^ signaling in DU145 (**A**) and 22Rv1 (**B**) cell lines. Addition of rWnt5a (10 µg/ml) indicated by arrows.
(TIF)
