# Supplementary material for: Elevated Level of Wnt5a Protein in Localized Prostate Cancer Tissue Is Associated with Better Outcome
Source: PLoS One. 2011 Oct 24;6(10):e26539. doi: 10.1371/journal.pone.0026539 (PMC3200334; doi:10.1371/journal.pone.0026539)
Supplement: Materials and Methods S1 — (DOC) [file pone.0026539.s001.doc]

**Supplementary – Materials and Methods**

**Immunocytochemistry of prostate cancer cell lines**

Immunocytochemistry was performed on LNCaP, 22Rv1 and DU145 cells. Cells were grown on glass coverslips coated with BD cell-Tak (354240, BD Biosciences, Bedford, MA) in a 24-well plate. Cells were transfected in the same way as described in the Materials & Methods section. Twenty-four hours after transfection cells were washed twice with PBS, fixed in 4% paraformaldehyde and cell membranes were permeabilized with 0.3% Triton X-100 in PBS. After peroxidase blocking in 3% H_2_O_2_-50% methanol in PBS coverslips were stained with Wnt5a ab overnight at 4°C. Coverslips were then washed and incubated with Envision System Labeled polymer-HRP anti-rabbit (Dako) for 30 min at room temperature. After incubation with DAB (Vector laboratories Inc) coverslips were counterstained with Mayers HTX, and dehydrated with series of increasing concentrations of ethanol. Coverslips were then mounted on glass slides with Pertex (00840, Histolab) after treatment in xylene for 5 min for complete removal of water.

**Measurement of calcium signaling**

Intracellular free calcium levels were measured in 22Rv1 and DU145 cells by loading the cells with fura-2. Briefly, cells were cultured in RPMI 1640 growth medium with 10% FBS, and serum starved for 24 hours before the experiment. Cells were trypsinized, washed and suspended in serum free medium. The cells were subsequently incubated at 37°C for 30 min with the fluorescent calcium indicator fura2-AM (4 µM), after which they were washed once and placed in a chamber containing a physiologically balanced calcium medium (136 mM NaCl, 4.7 mM KCl, 1.2 mM MgSO_4_, 1.1 mM CaCl_2_, 1.2 mM KH_2_PO_4_, 5 mM NaHCO_3_, 5.5 mM glucose, 20 mM HEPES) at 37 °C. Before and after stimulation with rWnt-5a (10µg/ml) cells were excited at 340 nm and 380 nm, and emission was measured at 510 nm. For each experiment the calcium response of at least 10 single cells was calculated as the change in fluorescence intensity ratio (340/380 nm).
