# Supplementary figures and images for: Elevated Level of Wnt5a Protein in Localized Prostate Cancer Tissue Is Associated with Better Outcome
Source: PLoS One. 2011 Oct 24;6(10):e26539. doi: 10.1371/journal.pone.0026539 (PMC3200334; doi:10.1371/journal.pone.0026539)

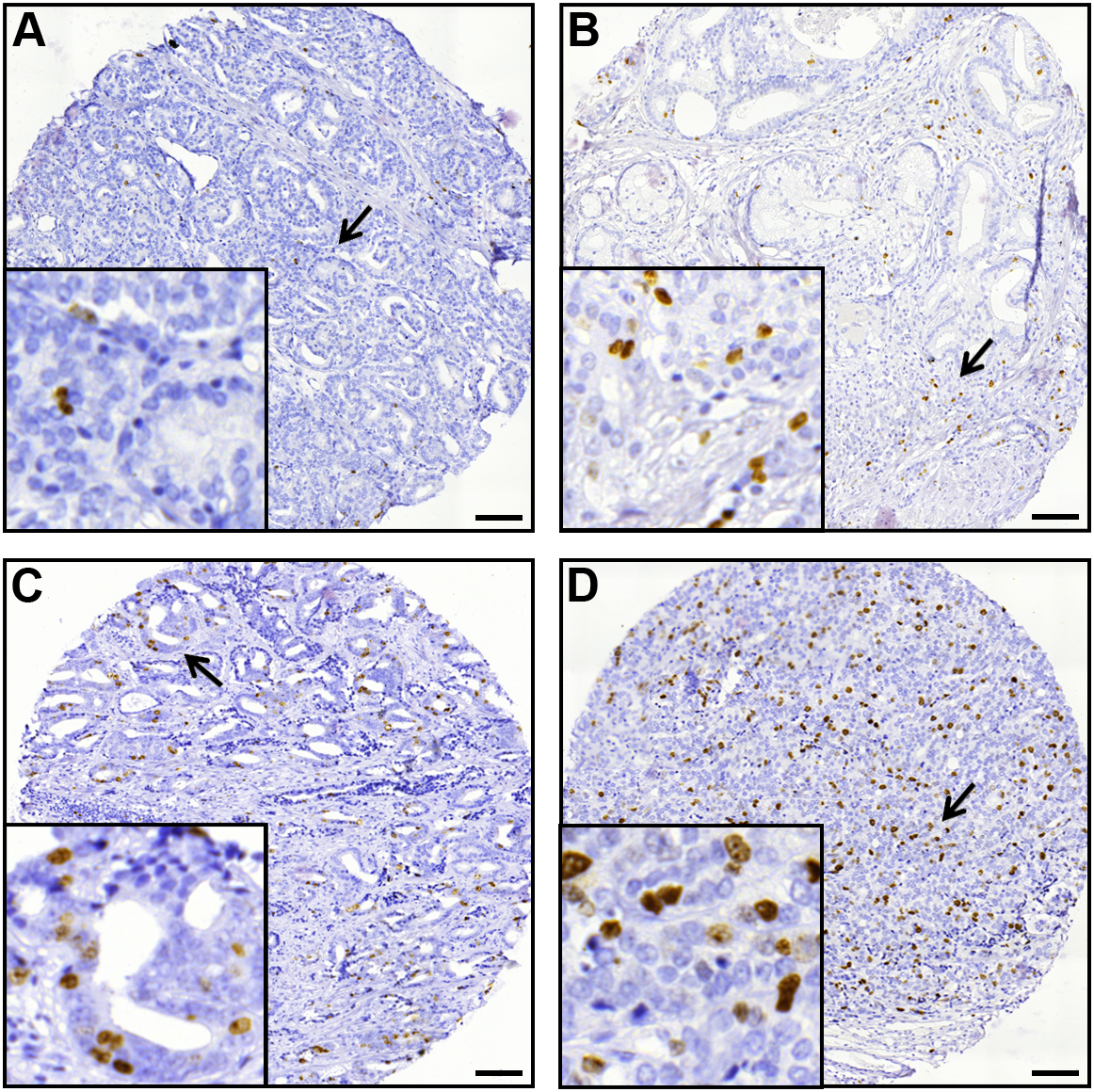

Supplement: Figure S1 — Representatives of Ki-67 nuclear fraction immunostainings. A) The panel represents cancer core with no Ki-67 nuclear staining. B) The panel represents cancer core with 1–3% Ki-67 nuclear staining, C) The panel shows cancer core with 4–10% of nuclei stained positive for Ki-67 D) The panel shows cancer core with more than 10% of nuclei stained positive for Ki-67. All inserts in the panels depict magnification (40×) images of the area indicated by the arrow in the larger image seen at 15× magnification. The bar in each panel outlines 100 µm. (TIF) [file pone.0026539.s002.tif]

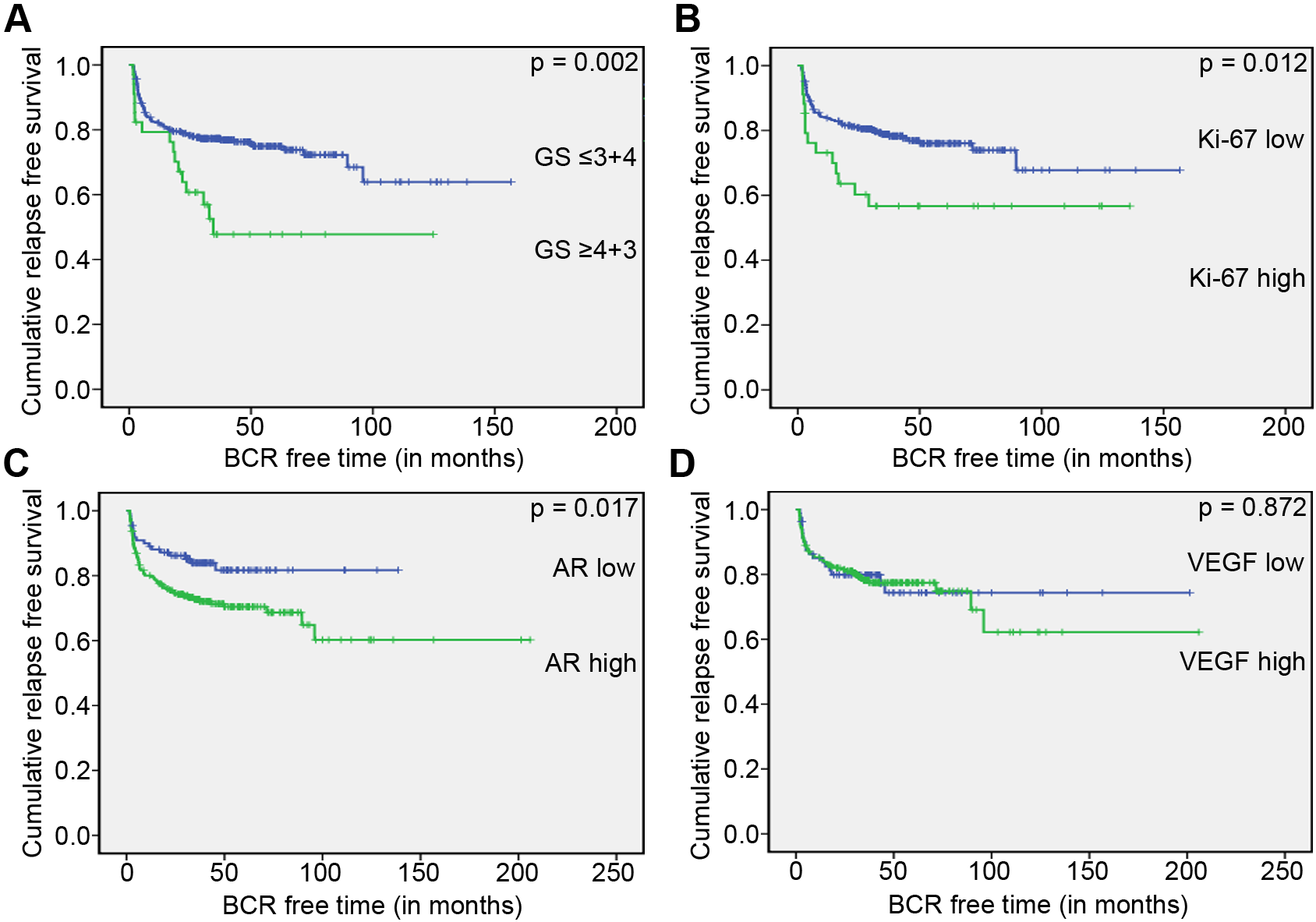

Supplement: Figure S2 — Validation of the patient material used in this study. A) The patient tumor material was divided into 2 groups based on their Gleason score (GS). As indicated in the panel one group had a Gleason score of ≤3+4 and the other a Gleason score of ≥4+3. Kaplan-Meier curves were then generated for each of the 2 groups with the indicated Gleason scores and their respective BCR free time. B) The panel shows Kaplan-Meier curves plotted between low or high Ki-67 expression and their respective BCR free time. C) The panel shows Kaplan-Meier curves plotted between low or high AR expression and their respective BCR free time. D) The panel shows Kaplan-Meier curves plotted between low or high VEGF expression and their respective BCR free time. (TIF) [file pone.0026539.s003.tif]

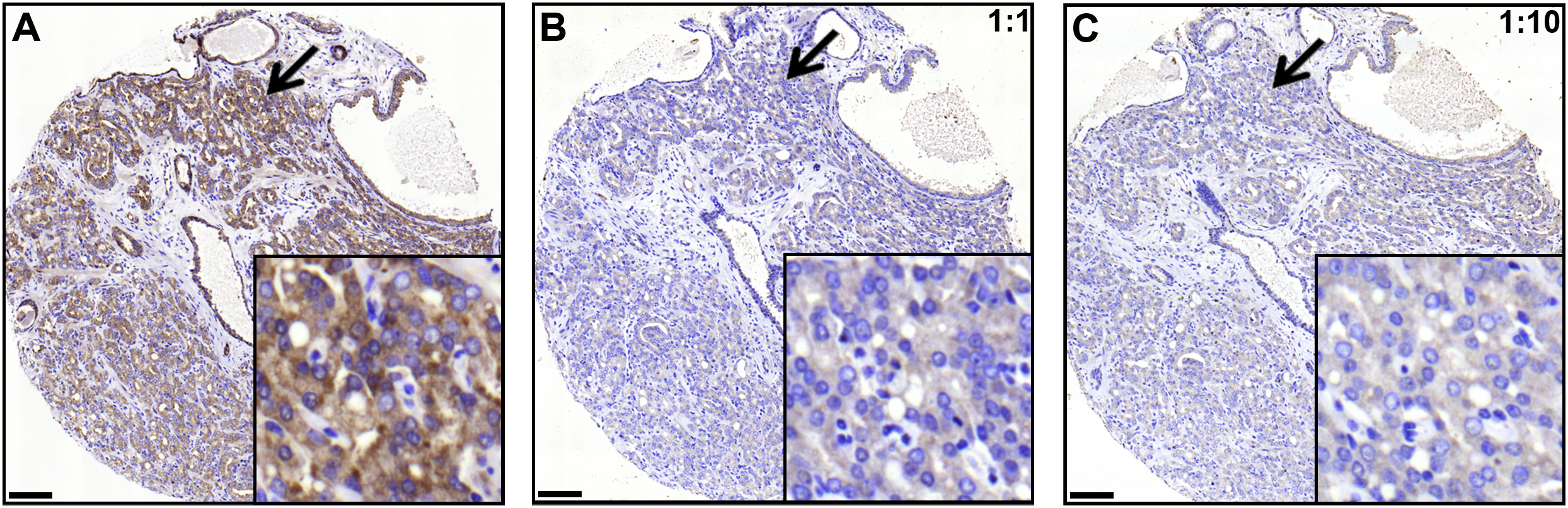

Supplement: Figure S3 — Validation of Wnt5a antibody specificity by blocking with rWnt5a. A shows a prostate cancer core section immunostained with anti-Wnt5a IgGs alone. B & C) Adjacent tissue sections immunostained using the same Wnt5a antibody after pre-incubated with rWnt5a at a molar ratio of 1∶1 or 1∶10, respectively. Each bar outlines 100 µm. (TIF) [file pone.0026539.s004.tif]

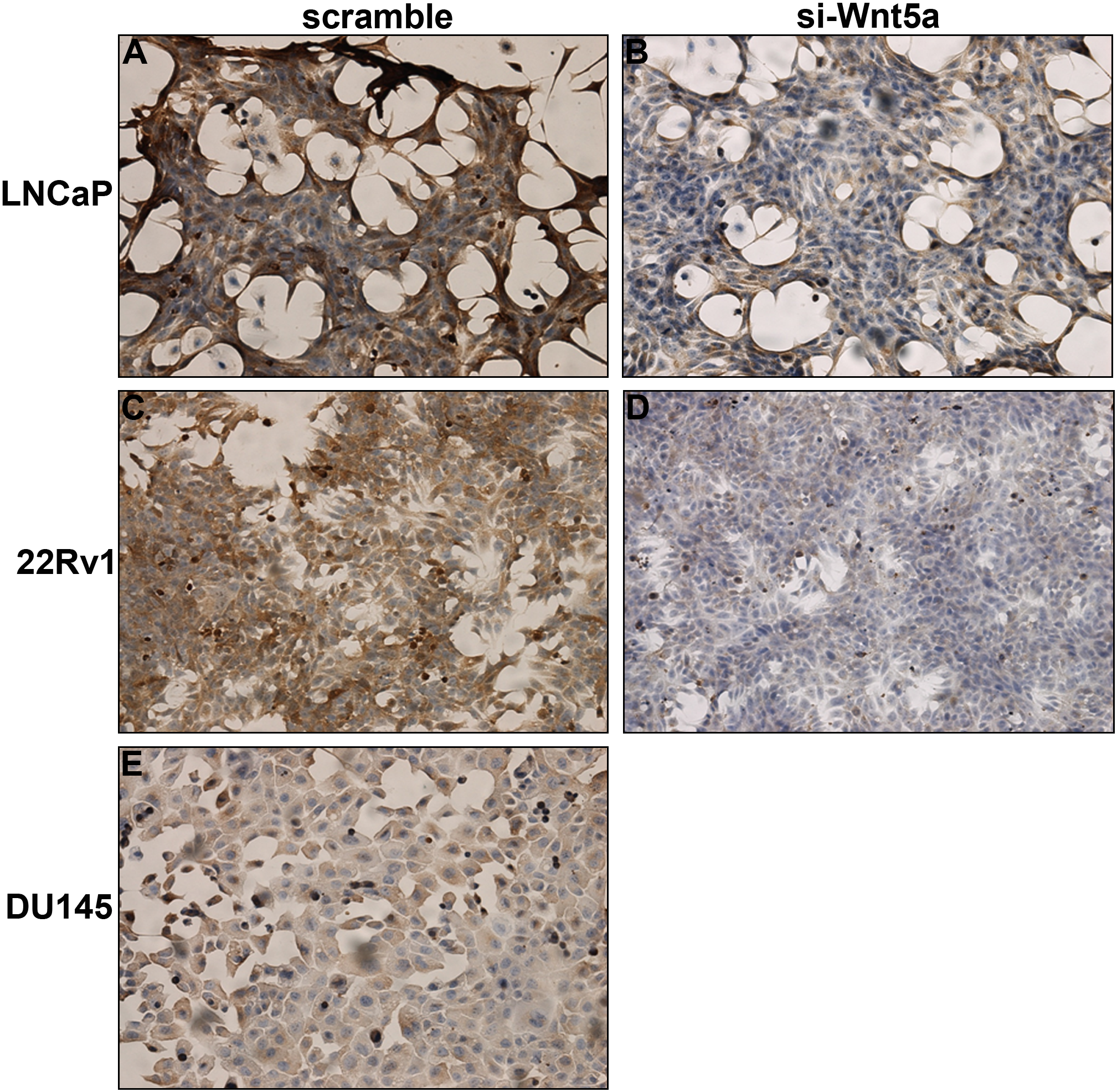

Supplement: Figure S4 — Immunocytochemistry of prostate cancer cell lines after Wnt5a knockdown using si-RNA, immunostained with Wnt5a antibody. A) Wnt5a staining in LNCaP cells transfected with scramble RNA. B) Decreased intensity of Wnt5a staining in LNCaP cells transfected with si-Wnt5a. C) Wnt5a staining of 22Rv1 cells transfected with scramble RNA. D) Decreased Wnt5a staining in 22Rv1 cells transfected with si-Wnt5a. E) Weak Wnt5a immunostaining in DU145 cells. (TIF) [file pone.0026539.s005.tif]

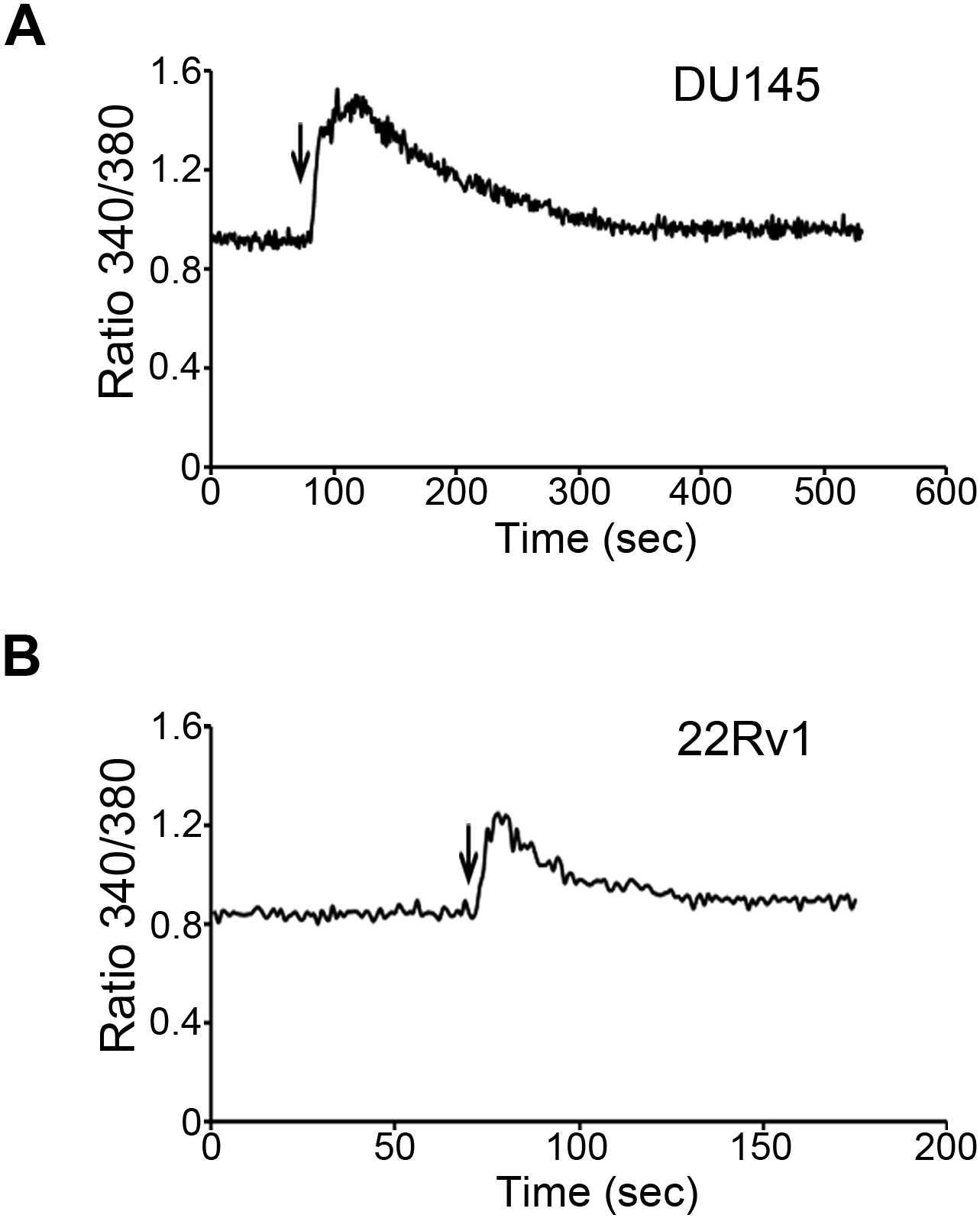

Supplement: Figure S5 — Measurement of intracellular Ca2+ signaling in DU145 (A) and 22Rv1 (B) cell lines. Addition of rWnt5a (10 µg/ml) indicated by arrows. (TIF) [file pone.0026539.s006.tif]
